# Real-world pharmacovigilance reports of hepatitis A inactivated and hepatitis B (recombinant) vaccine: insights from disproportionality analysis of the vaccine adverse event reporting system

**DOI:** 10.3389/fcimb.2025.1609409

**Published:** 2025-06-10

**Authors:** Yuhang Zhou, Yue Wang, Yun Feng, Huiyue Zhang, Tao Sun, Junnan Xu

**Affiliations:** ^1^ Department of Breast Medicine 1, Cancer Hospital of China Medical University, Liaoning Cancer Hospital, Shenyang, China; ^2^ Department of Pharmacology, Cancer Hospital of China Medical University, Liaoning Cancer Hospital, Shenyang, China; ^3^ Department of Breast Medicine, Cancer Hospital of Dalian University of Technology, Liaoning Cancer Hospital, Shenyang, China

**Keywords:** hepatitis AB vaccine, vaccine safety, surveillance, pharmacovigilance, VAERS

## Abstract

**Background:**

Hepatitis A Inactivated and Hepatitis B (Recombinant) Vaccine (Hep AB) was approved for use in 2001. Hep AB demonstrates satisfactory efficacy in protecting the public from hepatitis virus infections. However, there is a lack of recent real-world report on its adverse events (AEs).

**Methods:**

We retrieved US AE reports related to Hep AB vaccination from VAERS for the period 2020-2024. We used four algorithms: Reporting Odds Ratio (ROR), Proportional Reporting Ratio (PRR), Bayesian Confidence Propagation Neural Network (BCPNN) and Multi-Item Gamma-Poisson Shrinkage (MGPS) to examine AE signals. The ROR and PRR algorithms have higher sensitivity but lower specificity. However, BCPNN and MGPS compensate for this limitation. Combining all four algorithms helps reduce false-positive signals. In addition to the general population, we also focused on reports stratified by gender.

**Results:**

We retrieved 1,640 eligible reports from VAERS. In the general population, we identified two AE signals at the System Organ Classification (SOC) level. Additionally, we found 39 AE signals at the Preferred Term (PT) level. Among these, endocrine disorders were identified for the first time as AE signals. In the subsequent gender stratified analysis, more AE signals were identified in females compared to males. Notably, signals for endocrine disorders (autoimmune thyroiditis and Graves’ disease) were detected in females, whereas no such signals were found in males.

**Conclusions:**

We conducted a comprehensive examination of the recent AE reports for Hep AB and identified unexpected AEs, particularly in females. These findings will provide valuable insights into future evidence-based surveillance strategies of Hep AB.

## 1 Introduction

Hepatitis is a common liver disease, with viral hepatitis being one of its primary types (Cooke et al., 2024). Hepatitis A virus (HAV) and hepatitis B virus (HBV) are two important pathogens responsible for viral hepatitis. HAV is transmitted through the fecal-oral route, commonly through contaminated water, food or daily contact (Cuthbert, 2001). HBV is transmitted through blood and sexual contact (Daka et al., 2022; Fu et al., 2024). The symptoms of HAV infection manifest in the short term, including jaundice, abdominal pain and hyperbilirubinemia (Abutaleb and Kottilil, 2020). However, HBV infection causes chronic liver inflammation, which may eventually progress to cirrhosis and liver cancer (Trépo et al., 2014). In order to ensure public health, the HAV and HBV vaccines were introduced. They will prevent HAV and HBV infections by stimulating the body to produce specific antibodies. To meet broader health needs, the first Hepatitis A Inactivated and Hepatitis B (Recombinant) vaccine (Hep AB) was licensed for use in the United States in 2001 (Advisory Committee on Immunization Practices (ACIP) et al., 2006). Previous analysis showed that routine childhood vaccination would prevent 322 million cases of disease and approximately 732,000 premature deaths among children born between 1994 and 2013 (Hill et al., 2015). Viral hepatitis can also reduce its incidence through vaccination. The World Health Organization (WHO) has stated that there has been a notable decline in infection rates since the introduction of HAV and HBV vaccination (WHO position paper on hepatitis A vaccines: June 2012-recommendations, 2013; World Health Organization, 2019).

Like medications, vaccination can also induce a range of adverse events (AEs) and sometimes serious AEs (SAEs) (Li et al., 2024f; Zhang et al., 2024; Li et al., 2025e). Then, the safety of Hep AB is often a concern for vaccine recipients. The most commonly reported AEs in clinical trials were general fatigue, injection site pain, erythema and headache (Murdoch et al., 2003). Safety analysis of vaccination during pregnancy indicated that Hep AB vaccination did not show any patterns of adverse pregnancy outcomes (Celzo et al., 2020). Moreover, AEs after Hep AB vaccination were found to be similar to those reported after single-dose HAV and HBV vaccinations (Woo et al., 2006). These reports indicate a safety profile for Hep AB. The safety of Hep AB has been comprehensively evaluated prior to its market launch. Nevertheless, its long-term safety has still attracted widespread attention. Over the past decade, the safety of Hep AB vaccination has continued to be assessed. However, there has been a lack of real-world report of Hep AB vaccination in the past five years.

AE related studies in vaccine informatics have attracted significant public attention, as evidenced by recent research advancements (Li et al., 2024a, 2024b, 2025a; Pan et al., 2024; Huffman et al., 2025). These studies are essential for improving the safety monitoring of vaccines and understanding potential risks. One of the key systems used to monitor AEs is the Vaccine Adverse Event Reporting System (VAERS). This system plays a crucial role in collecting and analyzing reports of vaccine AEs, contributing to a better understanding of vaccine safety. VAERS is a passive reporting system, meaning that it relies on healthcare providers and the public to voluntarily report AEs (Li et al., 2024e, 2024c, 2024d, 2025e, 2025c, 2025b, 2025d; Moro et al., 2024; Oliveira et al., 2025). In this study, we aim to collect recent reports for Hep AB to identify AE signals. Four disproportionate analysis methods will be employed to examine AE signals. Besides the general population, we also focused on reports fstratified by gender. Overall, this study will provide valuable insights for the future evidence-based surveillance strategies of Hep AB.

## 2 Method

The data in VAERS includes demographic details of the vaccinated individuals, types of vaccines administered and information on AE reports (Shimabukuro et al., 2015). AEs and their related signs and symptoms are coded using the Medical Dictionary for Regulatory Activities (MedDRA, version 27.0). Each report may include one or more MedDRA Preferred Terms (PTs). PTs are not necessarily medically confirmed diagnoses. This study adheres to the REporting of A Disproportionality Analysis for DrUg Safety Signal Detection Using Individual Case Safety Reports in PharmacoVigilance (READUS-PV) guidelines (Fusaroli et al., 2024).

### 2.1 Data source and processing

We extracted AE reports submitted to VAERS from 2020 to 2024 in the US (https://vaers.hhs.gov/). Then, we conducted the following procedures to process the data: (1) We ensured that all reports originated from the US by excluding entries labeled as “Foreign”; (2) Duplicate reports were excluded; (3) We filtered the target vaccine by restricting the “VAX_NAME” field to “HEP A + HEP B”; (4) We also excluded entries labeled as “NO BRAND NAME”; (5) Reports of vaccination errors (such as incomplete vaccination courses) without describing AEs were excluded. All other AE reports in VAERS will be used as the reference group for comparison. All PTs will be mapped to their respective System Organ Classifications (SOCs) for further exploration.

### 2.2 Statistical analysis

Disproportionality analysis has been widely used in pharmacovigilance to detect AE signals. This method detects signals by comparing the difference in the occurrence frequency of specific event combinations with the background frequency. The 2 × 2 contingency table used in the analysis is detailed in **Supplementary Table 1**. The total for each PT or SOC constitutes the value of “A”. Moreover, the values of “B”, “C” and “D” are calculated for all PTs or SOCs based on different vaccines. Reporting Odds Ratio (ROR) and Proportional Reporting Ratio (PRR) are classified as frequency-based methods, indicating high sensitivity but low specificity. Bayesian Confidence Propagation Neural Network (BCPNN) and Multi-Item Gamma-Poisson Shrinkage (MGPS) are Bayesian algorithms capable of handling complex data, but they exhibit lower sensitivity in data detection. To reduce the false positive rate of AE signals, an AE signal is generated when all four methods detect a positive result. **Supplementary Table 2** presents the four algorithms and their respective thresholds for positive results.

## 3 Results

### 3.1 Descriptive characteristics

**Figure 1:**
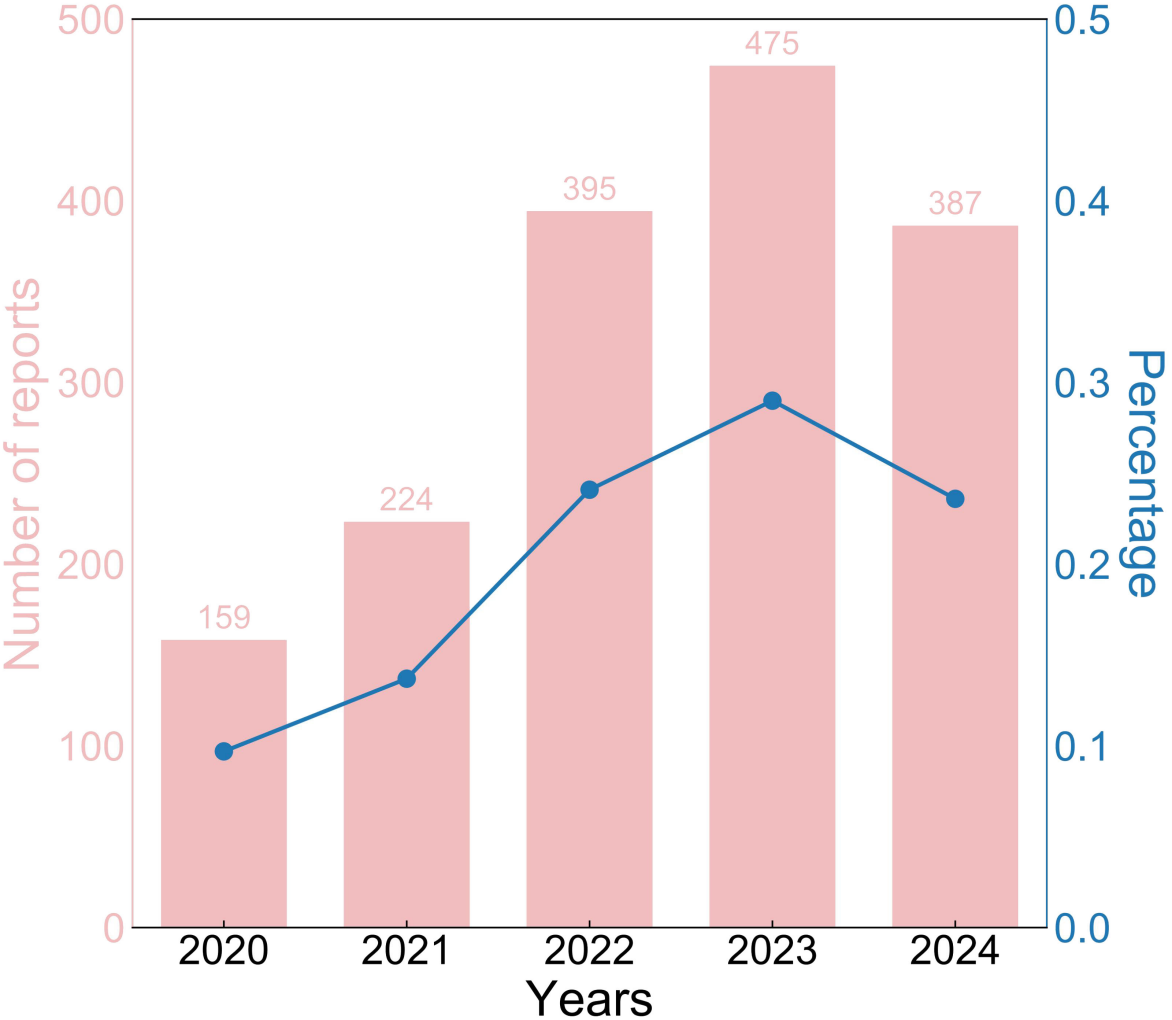
The historical trend of Hep AB-related AE reports from 2020 to 2024. Hep AB, Hepatitis A Inactivated and Hepatitis B (Recombinant) Vaccine; AE, adverse event.

A total of 1,640 reports were retrieved during the period from 2020 to 2024 (**Table 1**). Compared to males (38.9%), the majority of reports came from females (50.1%), with the remaining reports not specifying gender. Reports from the 18-64 age (41.0%) were significantly higher than those from other age. However, 696 reports (42.4%) did not record the age. Non-serious reports accounted for the majority (91.2%). Subsequently, we will conduct further exploration of the population with serious reports (8.8%). The majority of reports indicated vaccination with a single dose (72.4%). In addition, only five reports indicated deaths following vaccination. The number of life-threatening events occurring after vaccination increased to 26 cases. There have been 103 reports of hospitalizations due to vaccination. However, only one report indicated vaccination prolonged hospital stay. There were 37 reports indicating disability caused by vaccination. Regarding reporting years, there were 159 reports in 2020, 224 reports in 2021, 395 reports in 2022, 475 reports in 2023 and 387 reports in 2024. The historical trend in the number of reports is depicted in **Figure 1**.

**Table 1:** Clinical characteristics of Hep AB-related reports in the VAERS database.

Characteristics	Case Number (n)	Proportion (%)
Overall (TWINRIX)	1640	100
Gender		
Female	821	50.1
Male	638	38.9
Unknow	181	11.0
Age		
<18	66	4.0
18-64	673	41.0
65-84	195	11.9
>85	10	0.6
Unknow	696	42.4
Serious^a^		
No	1496	91.2
Yes	144	8.8
Alone^b^		
No	452	27.6
Yes	1188	72.4
Died^c^		
NotAvailable	1635	99.7
Yes	5	0.3
L_therapy^d^		
NotAvailable	1614	98.4
Yes	26	1.6
Hospital^e^		
NotAvailable	1537	93.7
Yes	103	6.3
X_stay^f^		
NotAvailable	1639	99.9
Yes	1	0.1
Disable		
NotAvailable	1603	97.7
Yes	37	2.3

a Developer classifies the adverse event as a serious report.

b Hep AB vaccine is administered as a standalone vaccination.

c Reports of deaths following vaccination.

d Life-threatening events related to vaccination occurred.

e Reports of hospitalization due to vaccination.

f Adverse events related to vaccination prolonged hospital stay.

Hep AB, Hepatitis A Inactivated and Hepatitis B (Recombinant) Vaccine; VAERS, Vaccine Adverse Event Reporting System.

Subsequently, we compiled a total of 1,138 reports with precise onset times of AEs (**Table 2**). The majority of reports (93.8%) indicated that the onset times of AEs within the first month following vaccination. However, AEs were still reported one year after vaccination (1.9%).

**Table 2:** Time to onset of AEs related to Hep AB vaccination.

Group	N	Proportion (%)
0-30 days	1068	93.8
31-60 days	22	1.9
61-90 days	8	7.0
91-120 days	3	3.0
121-150 days	2	0.2
151-180 days	1	0.1
181-360 days	12	1.1
>360 days	22	1.9

AEs, adverse events; Hep AB, Hepatitis A Inactivated and Hepatitis B (Recombinant) Vaccine.

### 3.2 Disproportionality analysis

#### 3.2.1 General population

To reduce the false positive rate, we only examined AE signals where all four algorithms met the threshold. We identified two AE signals at the SOC level in the general population (**Table 3**), including immune system disorders and endocrine disorders. Immune system disorders have already been reported in the package insert. However, endocrine disorders are being reported for the first time.

**Table 3:** The AE signals of Hep AB at the SOC level in general population.

SOC	A	ROR (95% CI)	PRR (χ2)	EBGM (EBGMO5)	IC (IC025)
Immune system disorders	68	3.31 (2.61-4.21)	3.28 (108.03)	3.28 (2.68)	1.71 (1.36)
Endocrine disorders	30	6.82 (4.76-9.78)	6.79 (147.57)	6.76 (5.01)	2.76 (2.24)

“A” represents the number of target adverse reaction reports in the 2 × 2 contingency table. AE, adverse event; Hep AB, Hepatitis A Inactivated and Hepatitis B (Recombinant) Vaccine; SOC, System Organ Class in MedDRA; ROR, Reporting Odds Ratio; PRR, Proportional Reporting Ratio; EBGM, Empirical Bayesian Geometric Mean; IC, Information Component.

**Figure 2:**
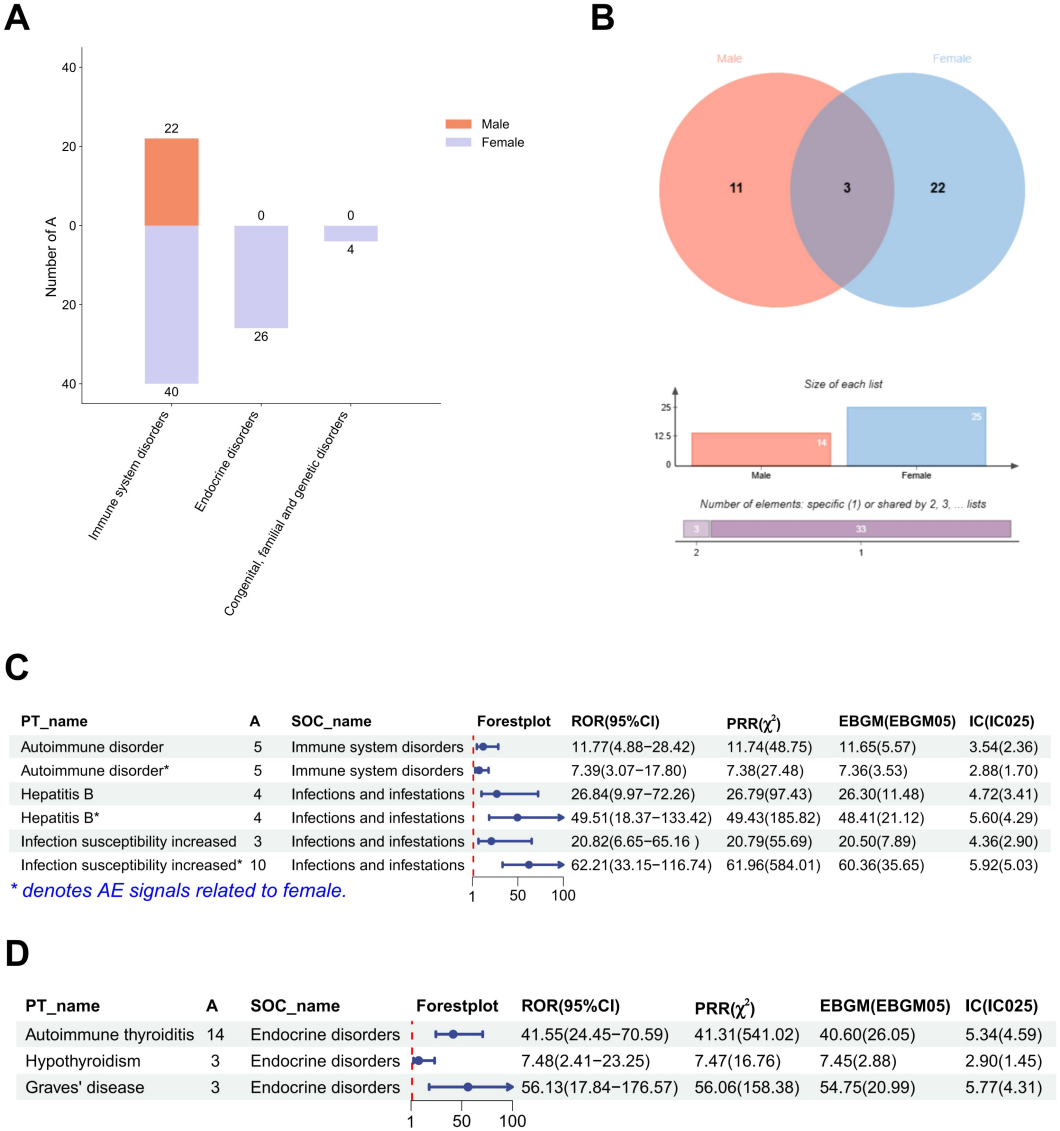
Gender stratified differences in AE signals at the SOC and PT levels. **(A)** The report number of AE signals at the SOC level. **(B)** Differences in AE signals between males and females at the PT level. **(C)** The strength of AE signals at the SOC level in males and females. **(D)** The strength of AE signals at the PT level under endocrine disorders in females. **(A)** represents the number of target adverse reaction reports in the 2 × 2 contingency table. PT, Preferred Term; SOC, System Organ Classification; AE, adverse event; ROR, Reporting Odds Ratio; PRR, Proportional Reporting Ratio; EBGM, Empirical Bayesian Geometric Mean; IC, Information Component.

After excluding SOCs and their PT signals unrelated to vaccination (including Injury, poisoning and procedural complications, General disorders and administration site conditions, Investigations, Product issues, and Social circumstances), 39 AE signals were detected at the PT level (**Table 4**). Under endocrine disorders, two PTs were identified as AE signals, including Autoimmune thyroiditis (ROR = 31.65; PRR = 31.54; EBGM05 = 20.50; IC025 = 4.25) and Graves’ disease (ROR = 33.80; PRR = 33.78; EBGM05 = 12.76; IC025 = 3.59) (**Figure 2**). **Supplementary Table 3** presents detailed information on the PT-level AE signals.

**Table 4:** The AE signals of Hep AB at the PT level in general population.

PT	A	ROR (95% CI)	PRR (χ2)	EBGM (EBGMO5)	IC (IC025)
Loss of consciousness	41	2.85 (2.10-3.88)	2.83 (48.74)	2.83 (2.19)	1.50 (1.05)
Unresponsive to stimuli	19	4.49 (2.86-7.05)	4.48 (51.25)	4.47 (3.06)	2.16 (1.51)
Autoimmune thyroiditis	16	31.65 (19.29-51.92)	31.54 (465.17)	31.02 (20.50)	4.96 (4.25)
Infection susceptibility increased	13	40.96 (23.62-71.03)	40.85 (494.4)	39.98 (25.23)	5.32 (4.54)
Autoimmune disorder	11	8.88 (4.91-16.08)	8.87 (76.41)	8.83 (5.37)	3.14 (2.31)
Hepatitis B	10	35.92 (19.2-67.21)	35.84 (332.23)	35.17 (20.82)	5.14 (4.26)
Seasonal allergy	9	29.98 (15.51-57.96)	29.92 (247.56)	29.46 (16.97)	4.88 (3.96)
Food allergy	6	15.38 (6.88-34.36)	15.36 (79.87)	15.24 (7.78)	3.93 (2.83)
Multiple sclerosis	6	7.09 (3.18-15.81)	7.08 (31.22)	7.06 (3.61)	2.82 (1.73)
Circulatory collapse	6	4.60 (2.06-10.25)	4.59 (16.83)	4.58 (2.34)	2.20 (1.10)
Acute disseminated encephalomyelitis	6	25.82 (11.53-57.82)	25.79 (140.98)	25.44 (12.96)	4.67 (3.57)
Skin exfoliation	6	5.09 (2.28-11.36)	5.09 (19.66)	5.08 (2.60)	2.34 (1.25)
Angiofibroma	5	3061.87 (731.51-12816.09)	3058.53 (5731.00)	1147.57 (346.36)	10.16 (8.64)
Neurological symptom	5	4.90 (2.04-11.79)	4.9 (15.46)	4.89 (2.34)	2.29 (1.11)
Resting tremor	4	204.08 (72.61-573.60)	203.9 (726.87)	183.61 (77.33)	7.52 (6.15)
Colitis	4	8.30 (3.11-22.18)	8.29 (25.55)	8.26 (3.63)	3.05 (1.75)
Encephalitis	4	6.73(2.52-17.96)	6.72 (19.41)	6.70 (2.95)	2.74 (1.45)
Personality change	4	27.62 (10.29-74.16)	27.6 (101.01)	27.2 (11.9)	4.77 (3.46)
Demyelination	4	14.87 (5.56-39.8)	14.86 (51.29)	14.75 (6.47)	3.88 (2.58)
Fibromyalgia	4	4.79 (1.79-12.78)	4.79 (11.95)	4.78 (2.10)	2.26 (0.96)
Immune system disorder	4	4.91 (1.84-13.10)	4.90 (12.40)	4.89 (2.15)	2.29 (1.00)
Hypotonic-hyporesponsive episode	4	5.47 (2.05-14.61)	5.47 (14.57)	5.46 (2.40)	2.45 (1.15)
Quadriparesis	3	24.06 (7.70-75.17)	24.04 (65.39)	23.74 (9.15)	4.57 (3.11)
Respiratory tract infection	3	8.99 (2.89-27.95)	8.98 (21.17)	8.94 (3.46)	3.16 (1.71)
Rhinitis	3	6.31 (2.03-19.61)	6.31 (13.35)	6.29 (2.43)	2.65 (1.21)
Neurodermatitis	3	22.04 (7.06-68.81)	22.02 (59.49)	21.77 (8.40)	4.44 (2.99)
Papule	3	8.93 (2.87-27.77)	8.92 (21.00)	8.88 (3.44)	3.15 (1.70)
Pharyngitis	3	8.27 (2.66-25.72)	8.27 (19.08)	8.23 (3.19)	3.04 (1.59)
Ataxia	3	6.07 (1.95-18.85)	6.06 (12.64)	6.05 (2.34)	2.60 (1.15)
Migraine with aura	3	6.37 (2.05-19.79)	6.36 (13.52)	6.35 (2.46)	2.67 (1.22)
Raynaud’s phenomenon	3	8.09 (2.60-25.15)	8.08 (18.54)	8.05 (3.12)	3.01 (1.56)
Seizure like phenomena	3	5.64 (1.82-17.54)	5.64 (11.42)	5.63 (2.18)	2.49 (1.05)
Eructation	3	7.86 (2.53-24.43)	7.85 (17.87)	7.82 (3.03)	2.97 (1.52)
Type 1 diabetes mellitus	3	6.79 (2.19-21.11)	6.79 (14.75)	6.77 (2.62)	2.76 (1.31)
Graves’ disease	3	33.8 (10.78-105.92)	33.78 (93.69)	33.18 (12.76)	5.05 (3.59)
Urticaria chronic	3	8.53 (2.74-26.52)	8.52 (19.83)	8.49 (3.28)	3.09 (1.64)
Tonsillitis	3	9.18 (2.95-28.56)	9.18 (21.75)	9.13 (3.53)	3.19 (1.74)
Peripheral vascular disorder	3	9.23 (2.97-28.7)	9.22 (21.88)	9.18 (3.55)	3.20 (1.75)
Leukopenia	3	8.22 (2.64-25.57)	8.22 (18.93)	8.18 (3.17)	3.03 (1.59)

“A” represents the number of target adverse reaction reports in the 2 × 2 contingency table. AE, adverse event; Hep AB, Hepatitis A Inactivated and Hepatitis B (Recombinant) Vaccine; PT, Preferred Term in MedDRA; ROR, Reporting Odds Ratio; PRR, Proportional Reporting Ratio; EBGM, Empirical Bayesian Geometric Mean; IC, Information Component.

#### 3.2.2 Gender stratification

After gender stratification, only immune system disorder was identified as AE signal in the male SOC level. However, three AE signals were identified at the SOC level in females, namely immune system disorders, endocrine disorders and congenital, familial and genetic disorders (**Table 5**). Reports of immune system disorders were fewer in males than in females (**Figure 2A**). A greater number of PT-level AE signals were detected in females. We examined the PT-level AE signals that were identified in both males and females, including autoimmune disorder, hepatitis B and increased infection susceptibility (**Figure 2B**). Except for increased infection susceptibility (Male: ROR = 20.82; PRR = 20.79; EBGM05 = 7.89; IC025 = 2.90 and Female: ROR = 62.21; PRR = 61.96; EBGM05 = 35.65; IC025 = 5.03), the signal strength of autoimmune disorder and hepatitis B did not show significant differences (**Figure 2C**). Subsequently, we conducted further investigation into the PT signals under the two SOC-level AE signals that were exclusive to females. Notably, congenital, familial and genetic disorders did not exhibit any PT-level AE signals. Three PT-level AE signals were identified under endocrine disorders, including autoimmune thyroiditis (ROR = 41.55; PRR = 41.31; EBGM05 = 26.05; IC025 = 4.59), hypothyroidism (ROR = 7.48; PRR = 7.47; EBGM05 = 2.88; IC025 = 1.45), and Graves’ disease (ROR = 56.13; PRR = 56.06; EBGM05 = 20.99; IC025 = 4.31) (**Figure 2D**). **Supplementary Tables 4** and **5** present detailed information on the PT-level AE signals for males and females, respectively.

**Table 5:** The AE signals of Hep AB at the SOC level under gender stratification.

SOC	A	ROR (95% CI)	PRR (χ2)	EBGM (EBGMO5)	IC (IC025)
Male					
Immune system disorders	22	3.43 (2.25-5.22)	3.4 (37.35)	3.4 (2.39)	1.76 (1.16)
Female					
Immune system disorders	40	1.32	3.47 (2.54-4.74)	3.43 (68.99)	3.42 (2.64)
Endocrine disorders	26	2.68	9.59 (6.51-14.13)	9.50 (197.03)	9.46 (6.84)
Congenital, familial and genetic disorders	4	1.1	5.28 (1.98-14.09)	5.27 (13.82)	5.26 (2.31)

“A” represents the number of target adverse reaction reports in the 2 × 2 contingency table. AE, adverse event; Hep AB, Hepatitis A Inactivated and Hepatitis B (Recombinant) Vaccine; SOC, System Organ Class in MedDRA; ROR, Reporting Odds Ratio; PRR, Proportional Reporting Ratio; EBGM, Empirical Bayesian Geometric Mean; IC, Information Component.

## 4 Discussion

In this study, we conducted a comprehensive analysis of AE signals following Hep AB vaccination using reports from VAERS between 2020 and 2024. Our findings indicate that the AEs observed following Hep AB vaccination are generally consistent with those listed in the package insert, including immune system disorders. In addition, we still identified unanticipated AEs, including endocrine disorders. Under gender stratification, no signals of endocrine disorders were found in males. In females, three PT-level AE signals under endocrine disorders (autoimmune thyroiditis, hypothyroidism, and Graves’ disease) were detected. Considering the continued administration of Hep AB, our findings will provide valuable insights for future evidence-based surveillance strategies of Hep AB.

Our findings suggest that females may be more susceptible to the occurrence of autoimmune thyroiditis and Graves’ disease following Hep AB vaccination. Both AEs are associated with autoimmune attacks. The signal strength is stronger in females, suggesting a potential association with gender. Gender, as a biological variable, influences the immune response to vaccination (Klein et al., 2010). Previous study has indicated that adult females exhibit stronger innate and adaptive immune responses compared to males (Klein and Flanagan, 2016). For example, plasmacytoid dendritic cells (pDCs) exposed to the TLR7 ligand *in vitro* resulted in higher production of interferon-α (IFN-α) in female cells compared to males (Berghöfer et al., 2006). In addition, following viral attack in adult rats, the expression of TLR pathways and pro-inflammatory genes was higher in the tissues of female rats compared to males (Hannah et al., 2008). Vaccines, as exogenous antigens, may activate a more sensitive immune response in females. Therefore, vaccination may exacerbate local or systemic inflammatory responses in females, potentially leading to autoimmune thyroiditis and Graves’ disease. Besides our research, studies on COVID-19 vaccine related AEs have also yielded similar findings (Li et al., 2024e, 2024c, 2025c, 2025b).

Epidemiological study indicates that the prevalence of autoimmune thyroid diseases in females (such as: Hashimoto’s thyroiditis and Graves’ disease) is significantly higher than in males (Vanderpump, 2011). Vaccination may act as a triggering factor, thereby enhancing susceptibility to autoimmune thyroiditis and Graves’ disease. A previous study has suggested that a positive result for anti-thyroid microsomal antibodies was observed after the second dose of the HAV vaccine (Karali et al., 2011). It is necessary to investigate whether the antigens of the hepatitis B vaccine (HBsAg) or the inactivated virus particles of the hepatitis A vaccine may have potential molecular mimicry or cross-reactivity with thyroid tissue, leading to immune system-induced attacks on thyroid cells.

In the reports we reviewed, 1,138 documented the complete onset time of vaccine-induced AEs. Almost all reports were submitted within the first month following vaccination. However, some reports indicated that AEs occurred one year after vaccination. Therefore, it is crucial to strengthen long-term surveillance of AEs following Hep AB vaccination.

In this study, although we identified unexpected AE signals, it must be acknowledged that there are certain limitations within this study. The AE signals obtained through disproportionality analysis did not include some of the common AEs in previous Hep AB clinical trial records. This suggests that future discussions on the safety of Hep AB vaccination should incorporate more real-world data and prospective evidence. Additionally, as VAERS is a passive reporting system, our results may have been influenced by the quality of AE reports, indicating that greater caution should be exercised when interpreting the findings. Finally, disproportionality analysis only detects AE signals and cannot be interpreted as indicating causality following vaccination. This requires further observational and experimental studies in the future to establish causality.

## 5 Conclusions

We conducted a comprehensive examination of the recent AE reports for Hep AB and identified unexpected AEs (autoimmune thyroiditis and Graves’ disease), particularly in females. These findings will provide valuable insights into future evidence-based surveillance strategies of Hep AB, ultimately facilitating more informed decisions in vaccine safety and public health policy.

## Data Availability

The original contributions presented in the study are included in the article/**Supplementary Material**. Further inquiries can be directed to the corresponding authors.
