# Supplementary material for: Real-world pharmacovigilance reports of hepatitis A inactivated and hepatitis B (recombinant) vaccine: insights from disproportionality analysis of the vaccine adverse event reporting system
Source: Front Cell Infect Microbiol. 2025 Jun 10;15:1609409. doi: 10.3389/fcimb.2025.1609409 (PMC12185504; doi:10.3389/fcimb.2025.1609409)
Supplement: Supplementary file 1 [file Table1.docx]

Supplementary Material

# Supplementary Tables

**Supplementary Table 1.** 2 × 2 contingency table for disproportionality analysis.

|  | **Number of target adverse reaction reports** | **Number of other adverse reaction reports** | **Total** |
| --- | --- | --- | --- |
| **Target drug** | A | B | A + B |
| **Other drugs** | C | D | C + D |
| **Total** | A + C | B + D | A + B + C + D |

**Supplementary Table 2.** Four major algorithms used for signal detection.

| Algorithms | Equation | Criteria |
| --- | --- | --- |
| ROR | ROR=ad/b/c | lower limit of 95% CI>1, N≥3 |
|  | 95%CI=e^ln(ROR)±1.96(1/a+1/b+1/c+1/d)^0.5^ |  |
| PRR | PRR=a(c+d)/c/(a+b) | PRR≥2, χ^2^≥4, N≥3 |
|  | χ^2^=[(ad-bc)^2](a+b+c+d)/[(a+b)(c+d)(a+c)(b+d)] |  |
| BCPNN | IC=log_2_a(a+b+c+d)(a+c)(a+b) | IC025>0 |
|  | 95%CI= E(IC) ± 2V(IC)^0.5 |  |
| MGPS | EBGM=a(a+b+c+d)/(a+c)/(a+b) | EBGM05>2 |
|  | 95%CI=e^ln(EBGM)±1.96(1/a+1/b+1/c+1/d)^0.5^ |  |

Equation: a, number of reports containing both the target drug and target adverse drug reaction; b, number of reports containing other adverse drug reaction of the target drug; c, number of reports containing the target adverse drug reaction of other drugs; d, number of reports containing other drugs and other adverse drug reactions. 95%CI, 95% confidence interval; N, the number of reports; χ^2^, chi-squared; IC, information component; IC025, the lower limit of 95% CI of the IC; E(IC), the IC expectations; V(IC), the variance of IC; EBGM, empirical Bayesian geometric mean; EBGM05, the lower limit of 95% CI of EBGM.

**Supplementary Table 3.** Detailed information on PT-level signals in the general population.

| **PT_name** | **SOC_name** |
| --- | --- |
| LOSS OF CONSCIOUSNESS | Nervous system disorders |
| UNRESPONSIVE TO STIMULI | Nervous system disorders |
| AUTOIMMUNE THYROIDITIS | Endocrine disorders |
| INFECTION SUSCEPTIBILITY INCREASED | Infections and infestations |
| AUTOIMMUNE DISORDER | Immune system disorders |
| HEPATITIS B | Infections and infestations |
| SEASONAL ALLERGY | Immune system disorders |
| FOOD ALLERGY | Immune system disorders |
| MULTIPLE SCLEROSIS | Nervous system disorders |
| CIRCULATORY COLLAPSE | Vascular disorders |
| ACUTE DISSEMINATED ENCEPHALOMYELITIS | Nervous system disorders |
| SKIN EXFOLIATION | Skin and subcutaneous tissue disorders |
| ANGIOFIBROMA | Neoplasms benign, malignant and unspecified (incl cysts and polyps) |
| NEUROLOGICAL SYMPTOM | Nervous system disorders |
| RESTING TREMOR | Nervous system disorders |
| COLITIS | Gastrointestinal disorders |
| ENCEPHALITIS | Infections and infestations |
| PERSONALITY CHANGE | Psychiatric disorders |
| DEMYELINATION | Nervous system disorders |
| FIBROMYALGIA | Musculoskeletal and connective tissue disorders |
| IMMUNE SYSTEM DISORDER | Immune system disorders |
| HYPOTONIC-HYPORESPONSIVE EPISODE | Nervous system disorders |
| QUADRIPARESIS | Nervous system disorders |
| RESPIRATORY TRACT INFECTION | Infections and infestations |
| RHINITIS | Infections and infestations |
| NEURODERMATITIS | Skin and subcutaneous tissue disorders |
| PAPULE | Skin and subcutaneous tissue disorders |
| PHARYNGITIS | Infections and infestations |
| ATAXIA | Nervous system disorders |
| MIGRAINE WITH AURA | Nervous system disorders |
| RAYNAUD'S PHENOMENON | Vascular disorders |
| SEIZURE LIKE PHENOMENA | Nervous system disorders |
| ERUCTATION | Gastrointestinal disorders |
| TYPE 1 DIABETES MELLITUS | Metabolism and nutrition disorders |
| GRAVES' DISEASE | Endocrine disorders |
| URTICARIA CHRONIC | Skin and subcutaneous tissue disorders |
| TONSILLITIS | Infections and infestations |
| PERIPHERAL VASCULAR DISORDER | Vascular disorders |
| LEUKOPENIA | Blood and lymphatic system disorders |

PT, Preferred Term; SOC, System Organ Classification.

**Supplementary Table 4.** The AE signals of Hep AB at the PT level in male.

| **PT** | **A** | **ROR (95% CI)** | **PRR (χ2)** | **EBGM (EBGMO5)** | **IC (IC025)** |
| --- | --- | --- | --- | --- | --- |
| SLEEP DISORDER | 13 | 3.98 ( 2.31 - 6.87 ) | 3.96 ( 28.74 ) | 3.95 ( 2.5 ) | 1.98 ( 1.21 ) |
| HYPERSENSITIVITY | 9 | 4.72 ( 2.45 - 9.1 ) | 4.7 ( 26.19 ) | 4.69 ( 2.71 ) | 2.23 ( 1.31 ) |
| ANGIOFIBROMA | 5 | 3491.35 ( 676.94 - 18006.86 ) | 3482.29 ( 4971.86 ) | 995.65 ( 252.34 ) | 9.96 ( 8.4 ) |
| AUTOIMMUNE DISORDER | 5 | 11.77 ( 4.88 - 28.42 ) | 11.74 ( 48.75 ) | 11.65 ( 5.57 ) | 3.54 ( 2.36 ) |
| HEPATITIS B | 4 | 26.84 ( 9.97 - 72.26 ) | 26.79 ( 97.43 ) | 26.3 ( 11.48 ) | 4.72 ( 3.41 ) |
| RESTING TREMOR | 4 | 398.8 ( 131.15 - 1212.67 ) | 397.98 ( 1231.94 ) | 309.76 ( 122.15 ) | 8.28 ( 6.82 ) |
| ENCEPHALITIS | 4 | 10.34 ( 3.86 - 27.67 ) | 10.32 ( 33.42 ) | 10.25 ( 4.5 ) | 3.36 ( 2.06 ) |
| PAPULE | 3 | 20.92 ( 6.69 - 65.49 ) | 20.89 ( 55.99 ) | 20.6 ( 7.93 ) | 4.36 ( 2.91 ) |
| PERSONALITY CHANGE | 3 | 34.59 ( 10.99 - 108.84 ) | 34.54 ( 95.33 ) | 33.72 ( 12.92 ) | 5.08 ( 3.61 ) |
| PHARYNGITIS | 3 | 16.61 ( 5.32 - 51.88 ) | 16.58 ( 43.42 ) | 16.4 ( 6.32 ) | 4.04 ( 2.58 ) |
| CHROMATURIA | 3 | 7.41 ( 2.38 - 23.05 ) | 7.4 ( 16.51 ) | 7.36 ( 2.85 ) | 2.88 ( 1.43 ) |
| SEIZURE LIKE PHENOMENA | 3 | 9.14 ( 2.93 - 28.46 ) | 9.12 ( 21.56 ) | 9.07 ( 3.51 ) | 3.18 ( 1.73 ) |
| SINUSITIS | 3 | 5.4 ( 1.74 - 16.79 ) | 5.39 ( 10.69 ) | 5.37 ( 2.08 ) | 2.43 ( 0.98 ) |
| INFECTION SUSCEPTIBILITY INCREASED | 3 | 20.82 ( 6.65 - 65.16 ) | 20.79 ( 55.69 ) | 20.5 ( 7.89 ) | 4.36 ( 2.9 ) |

“A” represents the number of target adverse reaction reports in the 2 × 2 contingency table. AE, adverse event; Hep AB, Hepatitis A Inactivated and Hepatitis B (Recombinant) Vaccine; PT, Preferred Term; PT, Preferred Term; ROR, Reporting Odds Ratio; PRR, Proportional Reporting Ratio; EBGM, Empirical Bayesian Geometric Mean; IC, Information Component.

**Supplementary Table 4.** The AE signals of Hep AB at the PT level in female.

| **PT** | **A** | **ROR (95% CI)** | **PRR (χ2)** | **EBGM (EBGMO5)** | **IC (IC025)** |
| --- | --- | --- | --- | --- | --- |
| LOSS OF CONSCIOUSNESS | 20 | 3.07 ( 1.97 - 4.76 ) | 3.05 ( 27.59 ) | 3.05 ( 2.11 ) | 1.61 ( 0.97 ) |
| AUTOIMMUNE THYROIDITIS | 14 | 41.55 ( 24.45 - 70.59 ) | 41.31 ( 541.02 ) | 40.6 ( 26.05 ) | 5.34 ( 4.59 ) |
| UNRESPONSIVE TO STIMULI | 11 | 6.49 ( 3.59 - 11.75 ) | 6.47 ( 50.73 ) | 6.45 ( 3.93 ) | 2.69 ( 1.85 ) |
| PALLOR | 10 | 3.78 ( 2.03 - 7.05 ) | 3.77 ( 20.36 ) | 3.77 ( 2.24 ) | 1.91 ( 1.04 ) |
| INFECTION SUSCEPTIBILITY INCREASED | 10 | 62.21 ( 33.15 - 116.74 ) | 61.96 ( 584.01 ) | 60.36 ( 35.65 ) | 5.92 ( 5.03 ) |
| SEASONAL ALLERGY | 7 | 44.09 ( 20.85 - 93.24 ) | 43.96 ( 288.39 ) | 43.15 ( 23.06 ) | 5.43 ( 4.4 ) |
| CIRCULATORY COLLAPSE | 5 | 7.53 ( 3.13 - 18.13 ) | 7.52 ( 28.16 ) | 7.5 ( 3.59 ) | 2.91 ( 1.72 ) |
| RESPIRATORY DISTRESS | 5 | 4.26 ( 1.77 - 10.25 ) | 4.25 ( 12.43 ) | 4.25 ( 2.04 ) | 2.09 ( 0.91 ) |
| AUTOIMMUNE DISORDER | 5 | 7.39 ( 3.07 - 17.8 ) | 7.38 ( 27.48 ) | 7.36 ( 3.53 ) | 2.88 ( 1.7 ) |
| HEPATITIS B | 4 | 49.51 ( 18.37 - 133.42 ) | 49.43 ( 185.82 ) | 48.41 ( 21.12 ) | 5.6 ( 4.29 ) |
| FOOD ALLERGY | 4 | 17.92 ( 6.69 - 47.96 ) | 17.89 ( 63.29 ) | 17.76 ( 7.79 ) | 4.15 ( 2.85 ) |
| MULTIPLE SCLEROSIS | 4 | 8.45 ( 3.16 - 22.57 ) | 8.44 ( 26.12 ) | 8.41 ( 3.69 ) | 3.07 ( 1.78 ) |
| ACUTE DISSEMINATED ENCEPHALOMYELITIS | 4 | 40.57 ( 15.08 - 109.12 ) | 40.5 ( 151.45 ) | 39.82 ( 17.4 ) | 5.32 ( 4.01 ) |
| SKIN EXFOLIATION | 4 | 6.25 ( 2.34 - 16.7 ) | 6.25 ( 17.58 ) | 6.23 ( 2.74 ) | 2.64 ( 1.35 ) |
| DIABETES MELLITUS | 3 | 10.56 ( 3.39 - 32.84 ) | 10.54 ( 25.8 ) | 10.5 ( 4.06 ) | 3.39 ( 1.94 ) |
| HYPOTHYROIDISM | 3 | 7.48 ( 2.41 - 23.25 ) | 7.47 ( 16.76 ) | 7.45 ( 2.88 ) | 2.9 ( 1.45 ) |
| COLITIS | 3 | 12.01 ( 3.86 - 37.36 ) | 11.99 ( 30.07 ) | 11.94 ( 4.62 ) | 3.58 ( 2.13 ) |
| ALTERED STATE OF CONSCIOUSNESS | 3 | 6.78 ( 2.18 - 21.08 ) | 6.77 ( 14.72 ) | 6.76 ( 2.62 ) | 2.76 ( 1.31 ) |
| HYPOTONIA | 3 | 5.3 ( 1.71 - 16.47 ) | 5.3 ( 10.43 ) | 5.29 ( 2.05 ) | 2.4 ( 0.96 ) |
| GRAVES' DISEASE | 3 | 56.13 ( 17.84 - 176.57 ) | 56.06 ( 158.38 ) | 54.75 ( 20.99 ) | 5.77 ( 4.31 ) |
| IMMUNE SYSTEM DISORDER | 3 | 7.64 ( 2.46 - 23.77 ) | 7.64 ( 17.25 ) | 7.61 ( 2.95 ) | 2.93 ( 1.48 ) |
| SKIN LESION | 3 | 5.36 ( 1.73 - 16.66 ) | 5.36 ( 10.61 ) | 5.35 ( 2.07 ) | 2.42 ( 0.97 ) |
| APATHY | 3 | 11.66 ( 3.75 - 36.29 ) | 11.65 ( 29.06 ) | 11.59 ( 4.48 ) | 3.54 ( 2.09 ) |
| PERIPHERAL VASCULAR DISORDER | 3 | 17.17 ( 5.51 - 53.51 ) | 17.15 ( 45.3 ) | 17.03 ( 6.58 ) | 4.09 ( 2.64 ) |
| LEUKOPENIA | 3 | 17 ( 5.46 - 52.98 ) | 16.98 ( 44.8 ) | 16.87 ( 6.52 ) | 4.08 ( 2.63 ) |

“A” represents the number of target adverse reaction reports in the 2 × 2 contingency table. AE, adverse event; Hep AB, Hepatitis A Inactivated and Hepatitis B (Recombinant) Vaccine; PT, Preferred Term; PT, Preferred Term; ROR, Reporting Odds Ratio; PRR, Proportional Reporting Ratio; EBGM, Empirical Bayesian Geometric Mean; IC, Information Component.
